# *N*,*N*′-Diethyl-*N*,*N*′-diphenyl­pyridine-2,6-dicarboxamide

**DOI:** 10.1107/S1600536812009026

**Published:** 2012-03-17

**Authors:** Blanka Klepetářová, Emanuel Makrlík, Vasily A. Babain, Václav Kašička

**Affiliations:** a Institute of Organic Chemistry and Biochemistry, Academy of Sciences of the Czech Republic, Flemingovo sq. 2, 166 10 Prague 6, Czech Republic; b Faculty of Environmental Sciences, Czech University of Life Sciences, Prague, Kamýcká 129, 165 21 Prague 6, Czech Republic; c Khlopin Radium Institute, Research and Production Association, 2nd Murinskiy Prospect b. 28, 194021 St Petersburg, Russian Federation

## Abstract

The asymmetric unit of the title compound, C_23_H_23_N_3_O_2_, contains two mol­ecules in both of which, one amide N atom is in a *syn* position with respect to the pyridine N atom, while the other amide N atom is in an *anti* position (the *syn-*-*anti* conformation). There are minor conformational differences between the two mol­ecules, as reflected in the N_pyridine_—C—C—N_amide_ torsion angles of −44.9 (3) and 136.0 (2)° for one mol­ecule and 43.5 (3) and −131.1 (2)° for the other mol­ecule. However, the two mol­ecules show significant differences in the orientation of an ethyl group, with corresponding C—C—N—C torsion angles of 86.6 (3)° for one mol­ecule and 79.6 (3)° for the other mol­ecule. In the crystal, mol­ecules are linked by weak C—H⋯O hydrogen bonds, forming a three-dimensional supra­molecular network.

## Related literature

For the extractive properties of some pyridine-dicarboxamides, see: Alyapyshev *et al.* (2004 ▶); Du Preez *et al.* (1987 ▶). For synthetic details, see: Shimada *et al.* (2004 ▶); Nikitskaya *et al.* (1958 ▶). For related structures, see: Malone *et al.* (1997 ▶); Fujiwara *et al.* (2008 ▶).
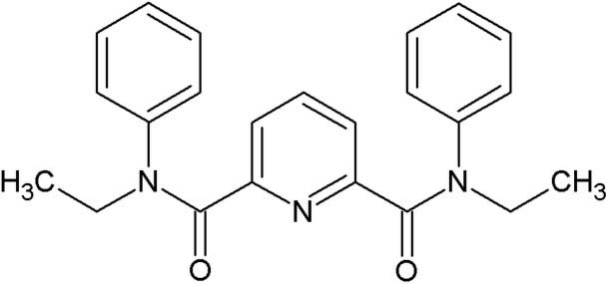


## Experimental

### 

#### Crystal data

C_23_H_23_N_3_O_2_
*M* *_r_* = 373.45
Triclinic, 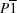

*a* = 12.1879 (17) Å
*b* = 12.2371 (15) Å
*c* = 13.6798 (17) Å
α = 83.971 (10)°
β = 86.919 (11)°
γ = 87.744 (10)°
*V* = 2024.9 (5) Å^3^
*Z* = 4
Cu *K*α radiation
μ = 0.63 mm^−1^
*T* = 170 K
0.37 × 0.19 × 0.15 mm

#### Data collection

Oxford Diffraction Xcalibur diffractometer
Absorption correction: multi-scan (*CrysAlis PRO*; Agilent, 2011 ▶) *T*_min_ = 0.496, *T*_max_ = 1.000
51216 measured reflections
8553 independent reflections
4586 reflections with *I* > 2σ(*I*)
*R*_int_ = 0.111

#### Refinement

*R*[*F* ^2^ > 2σ(*F* ^2^)] = 0.059
*wR*(*F* ^2^) = 0.062
*S* = 1.14
4586 reflections
505 parameters
H-atom parameters constrained
Δρ_max_ = 0.24 e Å^−3^
Δρ_min_ = −0.25 e Å^−3^

### 

Data collection: *CrysAlis PRO* (Agilent, 2011 ▶); cell refinement: *CrysAlis PRO*; data reduction: *CrysAlis PRO*; program(s) used to solve structure: *SIR92* (Altomare *et al.*, 1994 ▶); program(s) used to refine structure: *CRYSTALS* (Betteridge *et al.*, 2003 ▶); molecular graphics: *Mercury* (Macrae *et al.*, 2006 ▶); software used to prepare material for publication: *CRYSTALS*, *PLATON* (Spek, 2009 ▶) and *publCIF* (Westrip, 2010 ▶).

## Supplementary Material

Crystal structure: contains datablock(s) global, I. DOI: 10.1107/S1600536812009026/pv2514sup1.cif

Supplementary material file. DOI: 10.1107/S1600536812009026/pv2514Isup2.cdx

Structure factors: contains datablock(s) I. DOI: 10.1107/S1600536812009026/pv2514Isup3.hkl

Supplementary material file. DOI: 10.1107/S1600536812009026/pv2514Isup4.cml

Additional supplementary materials:  crystallographic information; 3D view; checkCIF report

## Figures and Tables

**Table 1:** Hydrogen-bond geometry (Å, °)

*D*—H⋯*A*	*D*—H	H⋯*A*	*D*⋯*A*	*D*—H⋯*A*
C34—H341⋯O1^i^	0.94	2.42	3.319 (4)	159
C42—H421⋯O2^ii^	0.94	2.43	3.271 (4)	150
C7—H71⋯O2^ii^	0.99	2.40	3.208 (4)	138
C43—H431⋯O3^iii^	0.95	2.66	3.443 (4)	140
C30—H301⋯O4^iv^	0.99	2.36	3.237 (4)	147
C23—H231⋯O4^v^	0.94	2.60	3.125 (3)	116

Symmetry codes: (i) 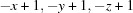; (ii) 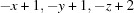; (iii) 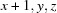; (iv) 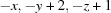; (v) 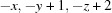.

**Fig. 1.**
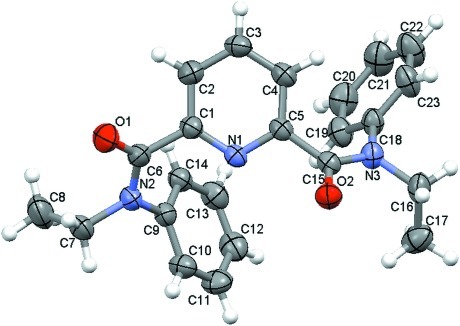
Molecule 1 of the title compound viewed perpendicular to the pyridine ring plane. Displacement ellipsoids are drawn at the 50% probability level, H atoms are shown as spheres of arbitrary radius.

**Fig. 2.**
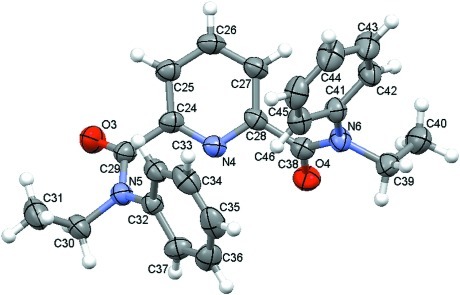
Molecule 2 of the title compound viewed perpendicular to the pyridine ring plane. Displacement ellipsoids are drawn at the 50% probability level, H atoms are shown as spheres of arbitrary radius.

**Fig. 3.**
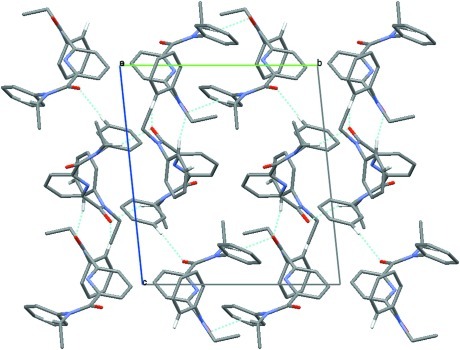
Projection along the a axis with highlighted hydrogen bonds between the molecules (H atoms not involved in hydrogen bonds were omitted for clarity).

## supplementary crystallographic information

### Comment

Pyridine-dicarboxamides have been studied recently for significant extractive properties (Alyapyshev *et al.*, 2004). The factors which lead to stabilization of low symmetry five-coordinate complexes, when using neutral donor ligands, have been reported (Du Preez *et al.*, 1987).

The title compound crystallizes with two independent molecules (1 and 2) (Figs. 1 & 2) in an asymmetric unit. The molecules consist of a pyridine ring between two amide groups in the *ortho* positions of this ring and ethyl and phenyl groups on the amide nitrogen. Both molecules in the asymmetric unit adopt a *syn*-anti conformation, in contrast to the *syn*-*syn* conformation found in *N,N'*-diphenylpyridine-2,6-dicarboxamide (Malone *et al.*, 1997), but analogous to that observed in *N,N'*-dimethyl-*N,N'*-diphenylpyridine-2,6-dicarboxamide (Fujiwara *et al.*, 2008).

There are minor conformational differences in the two molecules as reflected in the torsion angles N_pyridine_—*C*—*C*—N_amide_ being -44.9 (3) and 136.0 (2)° for molecule 1 and 43.5 (3) and -131.1 (2)° for molecule 2 and the torsion angles N_pyridine_—*C*—*C*—O_amide_ being 134.1 (3) and -46.8 (3)° for molecule 1 and -132.6 (3) and 51.2 (3)° for molecule 2. However, the two molecules show significant differences in the orientation of an ethyl group (the corresponding torsion angles are C17—C16—N3—C15 86.6 (3)° for molecule 1 and C40—C39—N6—C38 79.6 (3)° for molecule 2).

The molecules are connected *via* weak intermolecular C—H···O interactions between the amide oxygen atoms and ethyl and phenyl groups, forming a three-dimensional network (Fig. 3).

### Experimental

The title compound was synthesized as described in Shimada *et al.*, (2004), and Nikitskaya *et al.*, (1958). Colourless crystals were prepared by slow evaporation from acetonitrile.

### Refinement

The hydrogen atoms were located in in the Δρ map, but were repositioned geometrically. They were initially refined with soft restraints on the bond lengths and angles to regularize their geometry (C_methyl_—H = 0.96, C_methylene_—H = 0.97, C_aryl_—H = 0.93 Å) and fixed at those positions in the final cycles of refinements. The *U_iso_*(H) were allowed in the range 1.2–1.5 times *U_eq_* of the parent atom.

### Crystal data

**Table:**

C_23_H_23_N_3_O_2_	*Z* = 4
*M**_r_* = 373.45	*F*(000) = 792
Triclinic, *P*1	*D*_x_ = 1.225 Mg m^−^^3^
Hall symbol: -P 1	Cu *K*α radiation, λ = 1.54184 Å
*a* = 12.1879 (17) Å	Cell parameters from 6449 reflections
*b* = 12.2371 (15) Å	θ = 4.6–85.1°
*c* = 13.6798 (17) Å	µ = 0.63 mm^−^^1^
α = 83.971 (10)°	*T* = 170 K
β = 86.919 (11)°	Prism, colourless
γ = 87.744 (10)°	0.37 × 0.19 × 0.15 mm
*V* = 2024.9 (5) Å^3^	

### Data collection

**Table:**

Oxford Diffraction Xcalibur diffractometer	4586 reflections with *I* > 2σ(*I*)
Graphite monochromator	*R*_int_ = 0.111
φ & ω scans	θ_max_ = 85.7°, θ_min_ = 4.6°
Absorption correction: multi-scan (*CrysAlis PRO*; Agilent, 2011)	*h* = −15→14
*T*_min_ = 0.496, *T*_max_ = 1.000	*k* = −15→15
51216 measured reflections	*l* = −17→17
8553 independent reflections	

### Refinement

**Table:**

Refinement on *F*	Primary atom site location: structure-invariant direct methods
Least-squares matrix: full	Hydrogen site location: difference Fourier map
*R*[*F*^2^ > 2σ(*F*^2^)] = 0.059	H-atom parameters constrained
*wR*(*F*^2^) = 0.062	Method, part 1, Chebychev polynomial, [*w*eight] = 1.0/[A_0_*T_0_(x) + A_1_*T_1_(x) ··· + A_n-1_]*T_n-1_(x)] where A_i_ are the Chebychev coefficients listed belo*w* and x = *F* /*F*max Method = Robust Weighting W = [*w*eight] * [1-(delta*F*/6*sigma*F*)^2^]^2^ A_i_ are: 15.1 3.75 12.4 3.85
*S* = 1.14	(Δ/σ)_max_ = 0.003
4586 reflections	Δρ_max_ = 0.24 e Å^−^^3^
505 parameters	Δρ_min_ = −0.25 e Å^−^^3^
0 restraints	

### Fractional atomic coordinates and isotropic or equivalent isotropic displacement parameters (Å^2^)

**Table:**

	*x*	*y*	*z*	*U*_iso_*/*U*_eq_	
C1	0.4330 (2)	0.2215 (2)	0.95742 (19)	0.0386	
C2	0.4133 (2)	0.1145 (2)	0.9394 (2)	0.0470	
C3	0.3268 (2)	0.0603 (2)	0.9907 (2)	0.0466	
C4	0.2643 (2)	0.1148 (2)	1.0583 (2)	0.0419	
C5	0.2913 (2)	0.21987 (19)	1.07487 (18)	0.0360	
C6	0.5312 (2)	0.2762 (2)	0.9039 (2)	0.0419	
C7	0.6225 (2)	0.4370 (2)	0.8246 (2)	0.0498	
C8	0.6299 (3)	0.4396 (3)	0.7145 (2)	0.0742	
C9	0.4200 (2)	0.4453 (2)	0.86369 (18)	0.0389	
C10	0.4104 (2)	0.5463 (2)	0.9016 (2)	0.0498	
C11	0.3109 (3)	0.6049 (2)	0.8987 (2)	0.0580	
C12	0.2219 (3)	0.5641 (3)	0.8578 (2)	0.0575	
C13	0.2333 (2)	0.4658 (2)	0.8175 (2)	0.0526	
C14	0.3322 (2)	0.4063 (2)	0.81921 (19)	0.0425	
C15	0.2394 (2)	0.2773 (2)	1.15845 (19)	0.0375	
C16	0.0788 (2)	0.3366 (3)	1.2565 (2)	0.0514	
C17	0.0639 (3)	0.4588 (3)	1.2328 (3)	0.0633	
C18	0.0545 (2)	0.2535 (2)	1.10042 (19)	0.0418	
C19	0.0553 (2)	0.3071 (2)	1.0066 (2)	0.0507	
C20	−0.0191 (3)	0.2776 (3)	0.9415 (2)	0.0609	
C21	−0.0940 (3)	0.1977 (3)	0.9701 (2)	0.0662	
C22	−0.0940 (3)	0.1460 (3)	1.0641 (3)	0.0677	
C23	−0.0199 (2)	0.1736 (3)	1.1292 (2)	0.0544	
O1	0.61698 (16)	0.22033 (17)	0.89549 (17)	0.0626	
O2	0.29906 (15)	0.31442 (15)	1.21633 (13)	0.0468	
N3	0.12827 (17)	0.28338 (18)	1.17164 (15)	0.0424	
N1	0.37461 (16)	0.27412 (16)	1.02507 (15)	0.0357	
N2	0.52272 (17)	0.38349 (17)	0.86884 (16)	0.0418	
C24	0.0880 (2)	0.7024 (2)	0.49936 (19)	0.0397	
C25	0.1251 (2)	0.5935 (2)	0.4997 (2)	0.0451	
C26	0.2181 (2)	0.5602 (2)	0.5492 (2)	0.0496	
C27	0.2706 (2)	0.6350 (2)	0.5969 (2)	0.0444	
C28	0.2275 (2)	0.7416 (2)	0.59462 (18)	0.0380	
C29	−0.0175 (2)	0.7392 (2)	0.4509 (2)	0.0436	
C30	−0.1313 (2)	0.8812 (2)	0.3630 (2)	0.0513	
C31	−0.1468 (3)	0.8498 (3)	0.2607 (3)	0.0708	
C32	0.0714 (2)	0.9001 (2)	0.36448 (19)	0.0387	
C33	0.1532 (2)	0.8537 (2)	0.30747 (19)	0.0442	
C34	0.2459 (2)	0.9125 (3)	0.2747 (2)	0.0564	
C35	0.2549 (3)	1.0171 (3)	0.2993 (2)	0.0597	
C36	0.1719 (3)	1.0653 (2)	0.3539 (2)	0.0550	
C37	0.0792 (2)	1.0082 (2)	0.3859 (2)	0.0463	
C38	0.2727 (2)	0.8246 (2)	0.65339 (18)	0.0401	
C39	0.4275 (2)	0.9181 (2)	0.7089 (2)	0.0497	
C40	0.4483 (3)	0.8608 (3)	0.8096 (2)	0.0637	
C41	0.4545 (2)	0.8057 (2)	0.5676 (2)	0.0425	
C42	0.5530 (2)	0.7520 (2)	0.5924 (2)	0.0500	
C43	0.6267 (3)	0.7185 (3)	0.5201 (2)	0.0598	
C44	0.6015 (3)	0.7378 (3)	0.4228 (2)	0.0631	
C45	0.5031 (3)	0.7917 (3)	0.3978 (2)	0.0583	
C46	0.4301 (2)	0.8273 (2)	0.4697 (2)	0.0489	
O3	−0.09683 (16)	0.67804 (17)	0.46445 (18)	0.0632	
O4	0.21148 (15)	0.87033 (16)	0.71207 (14)	0.0519	
N4	0.13673 (16)	0.77617 (16)	0.54716 (16)	0.0382	
N5	−0.02370 (16)	0.83951 (17)	0.39990 (16)	0.0415	
N6	0.38214 (17)	0.84479 (18)	0.64277 (16)	0.0423	
H231	−0.0210	0.1386	1.1936	0.0654*	
H221	−0.1462	0.0916	1.0845	0.0811*	
H211	−0.1445	0.1790	0.9260	0.0788*	
H201	−0.0173	0.3119	0.8768	0.0734*	
H191	0.1060	0.3630	0.9869	0.0616*	
H161	0.1259	0.3218	1.3129	0.0612*	
H162	0.0068	0.3057	1.2741	0.0615*	
H173	0.0244	0.4913	1.2878	0.0944*	
H172	0.1344	0.4930	1.2207	0.0951*	
H171	0.0213	0.4736	1.1745	0.0948*	
H141	0.3408	0.3384	0.7909	0.0512*	
H131	0.1734	0.4373	0.7870	0.0622*	
H121	0.1543	0.6034	0.8558	0.0680*	
H111	0.3023	0.6723	0.9263	0.0698*	
H101	0.4709	0.5746	0.9310	0.0594*	
H71	0.6189	0.5137	0.8412	0.0594*	
H72	0.6863	0.3958	0.8527	0.0604*	
H83	0.6934	0.4796	0.6865	0.1108*	
H82	0.5637	0.4769	0.6886	0.1109*	
H81	0.6339	0.3658	0.6952	0.1106*	
H21	0.4576	0.0801	0.8922	0.0559*	
H31	0.3093	−0.0123	0.9805	0.0562*	
H41	0.2054	0.0799	1.0950	0.0511*	
H421	0.5689	0.7381	0.6588	0.0599*	
H431	0.6959	0.6849	0.5362	0.0718*	
H441	0.6513	0.7140	0.3732	0.0760*	
H451	0.4856	0.8042	0.3310	0.0698*	
H461	0.3646	0.8654	0.4525	0.0592*	
H391	0.3780	0.9818	0.7126	0.0601*	
H392	0.4953	0.9472	0.6771	0.0601*	
H403	0.4874	0.9106	0.8475	0.0956*	
H402	0.3775	0.8418	0.8441	0.0962*	
H401	0.4928	0.7931	0.8025	0.0955*	
H251	0.0875	0.5434	0.4674	0.0537*	
H261	0.2456	0.4864	0.5501	0.0607*	
H271	0.3332	0.6137	0.6301	0.0538*	
H331	0.1465	0.7825	0.2921	0.0536*	
H341	0.3009	0.8793	0.2351	0.0679*	
H351	0.3176	1.0570	0.2771	0.0718*	
H361	0.1779	1.1372	0.3689	0.0659*	
H371	0.0216	1.0420	0.4232	0.0553*	
H301	−0.1352	0.9625	0.3612	0.0615*	
H302	−0.1898	0.8524	0.4087	0.0618*	
H311	−0.2184	0.8768	0.2392	0.1055*	
H313	−0.0901	0.8829	0.2156	0.1060*	
H312	−0.1410	0.7692	0.2607	0.1061*	

### Atomic displacement parameters (Å^2^)

**Table:**

	*U*^11^	*U*^22^	*U*^33^	*U*^12^	*U*^13^	*U*^23^
C1	0.0370 (13)	0.0365 (13)	0.0422 (14)	−0.0022 (11)	−0.0021 (11)	−0.0034 (11)
C2	0.0490 (16)	0.0411 (15)	0.0518 (16)	−0.0016 (12)	0.0018 (13)	−0.0109 (12)
C3	0.0500 (16)	0.0362 (14)	0.0552 (17)	−0.0078 (12)	−0.0048 (14)	−0.0080 (12)
C4	0.0372 (14)	0.0418 (14)	0.0463 (15)	−0.0073 (11)	−0.0030 (12)	−0.0002 (12)
C5	0.0339 (13)	0.0349 (13)	0.0384 (13)	−0.0022 (10)	−0.0046 (11)	0.0014 (11)
C6	0.0373 (14)	0.0418 (15)	0.0480 (15)	−0.0032 (12)	0.0014 (12)	−0.0120 (12)
C7	0.0425 (15)	0.0523 (16)	0.0553 (17)	−0.0139 (13)	0.0059 (13)	−0.0093 (13)
C8	0.071 (2)	0.090 (3)	0.062 (2)	−0.031 (2)	0.0159 (18)	−0.0085 (18)
C9	0.0411 (14)	0.0384 (14)	0.0365 (13)	−0.0055 (11)	0.0037 (11)	−0.0027 (11)
C10	0.0523 (17)	0.0406 (15)	0.0574 (17)	−0.0071 (13)	−0.0044 (14)	−0.0057 (13)
C11	0.068 (2)	0.0381 (15)	0.067 (2)	0.0043 (14)	−0.0023 (16)	−0.0062 (14)
C12	0.0490 (17)	0.0528 (18)	0.067 (2)	0.0027 (14)	−0.0016 (15)	0.0064 (15)
C13	0.0479 (17)	0.0573 (18)	0.0521 (17)	−0.0078 (14)	−0.0087 (14)	0.0013 (14)
C14	0.0462 (15)	0.0427 (14)	0.0388 (14)	−0.0084 (12)	−0.0022 (12)	−0.0036 (11)
C15	0.0372 (14)	0.0351 (13)	0.0397 (14)	−0.0040 (11)	−0.0031 (12)	0.0002 (11)
C16	0.0459 (16)	0.0668 (19)	0.0413 (15)	−0.0031 (14)	0.0078 (13)	−0.0092 (14)
C17	0.0520 (18)	0.066 (2)	0.075 (2)	0.0078 (16)	−0.0089 (16)	−0.0252 (17)
C18	0.0321 (13)	0.0548 (16)	0.0390 (14)	−0.0035 (12)	−0.0009 (11)	−0.0063 (12)
C19	0.0468 (16)	0.0563 (17)	0.0468 (16)	−0.0020 (13)	−0.0008 (13)	0.0041 (13)
C20	0.0582 (19)	0.079 (2)	0.0453 (17)	0.0049 (17)	−0.0091 (15)	−0.0032 (15)
C21	0.0454 (17)	0.099 (3)	0.058 (2)	−0.0085 (18)	−0.0122 (15)	−0.0158 (19)
C22	0.0478 (18)	0.095 (3)	0.062 (2)	−0.0235 (18)	−0.0053 (16)	−0.0051 (18)
C23	0.0424 (15)	0.075 (2)	0.0447 (16)	−0.0145 (15)	−0.0021 (13)	0.0042 (14)
O1	0.0434 (11)	0.0531 (12)	0.0899 (16)	0.0060 (10)	0.0121 (11)	−0.0106 (11)
O2	0.0454 (11)	0.0521 (11)	0.0443 (10)	−0.0057 (9)	−0.0053 (9)	−0.0085 (9)
N3	0.0366 (12)	0.0524 (13)	0.0379 (12)	−0.0026 (10)	0.0004 (9)	−0.0042 (10)
N1	0.0316 (11)	0.0365 (11)	0.0387 (11)	−0.0043 (9)	−0.0029 (9)	−0.0009 (9)
N2	0.0364 (12)	0.0399 (12)	0.0492 (13)	−0.0060 (9)	0.0038 (10)	−0.0059 (10)
C24	0.0377 (14)	0.0335 (13)	0.0462 (15)	−0.0051 (11)	0.0063 (12)	0.0016 (11)
C25	0.0454 (15)	0.0366 (14)	0.0525 (16)	−0.0032 (12)	0.0006 (13)	−0.0021 (12)
C26	0.0493 (17)	0.0334 (14)	0.0635 (18)	0.0034 (12)	0.0046 (14)	0.0016 (13)
C27	0.0401 (14)	0.0397 (14)	0.0512 (16)	0.0030 (12)	−0.0020 (12)	0.0038 (12)
C28	0.0304 (13)	0.0411 (14)	0.0400 (14)	0.0019 (11)	0.0050 (11)	0.0027 (11)
C29	0.0354 (14)	0.0361 (14)	0.0598 (17)	−0.0027 (11)	−0.0029 (13)	−0.0066 (12)
C30	0.0409 (15)	0.0528 (17)	0.0599 (18)	0.0059 (13)	−0.0098 (14)	−0.0035 (14)
C31	0.057 (2)	0.090 (3)	0.068 (2)	0.0091 (18)	−0.0196 (17)	−0.0150 (19)
C32	0.0352 (14)	0.0394 (14)	0.0409 (14)	−0.0020 (11)	−0.0024 (11)	−0.0016 (11)
C33	0.0418 (15)	0.0526 (16)	0.0379 (14)	0.0072 (12)	−0.0043 (12)	−0.0059 (12)
C34	0.0431 (16)	0.077 (2)	0.0449 (16)	0.0075 (15)	0.0045 (13)	0.0064 (15)
C35	0.0513 (18)	0.070 (2)	0.0541 (18)	−0.0158 (16)	−0.0105 (15)	0.0211 (16)
C36	0.068 (2)	0.0455 (16)	0.0504 (17)	−0.0169 (15)	−0.0076 (15)	0.0072 (13)
C37	0.0547 (17)	0.0388 (14)	0.0441 (15)	−0.0022 (12)	0.0050 (13)	−0.0009 (12)
C38	0.0399 (15)	0.0416 (14)	0.0378 (14)	0.0021 (12)	0.0011 (12)	−0.0016 (11)
C39	0.0452 (16)	0.0491 (16)	0.0571 (17)	0.0001 (13)	−0.0043 (13)	−0.0162 (13)
C40	0.076 (2)	0.0602 (19)	0.0578 (19)	0.0018 (17)	−0.0152 (17)	−0.0145 (15)
C41	0.0374 (14)	0.0478 (15)	0.0421 (15)	−0.0052 (12)	−0.0010 (12)	−0.0033 (12)
C42	0.0441 (15)	0.0633 (18)	0.0442 (15)	0.0039 (14)	−0.0061 (13)	−0.0134 (13)
C43	0.0410 (16)	0.077 (2)	0.066 (2)	0.0029 (15)	−0.0003 (14)	−0.0306 (17)
C44	0.0497 (18)	0.083 (2)	0.059 (2)	−0.0124 (17)	0.0142 (15)	−0.0242 (17)
C45	0.061 (2)	0.076 (2)	0.0401 (16)	−0.0208 (17)	0.0019 (15)	−0.0080 (15)
C46	0.0440 (15)	0.0582 (17)	0.0440 (16)	−0.0073 (13)	−0.0012 (13)	−0.0012 (13)
O3	0.0422 (11)	0.0507 (12)	0.0963 (17)	−0.0131 (10)	−0.0086 (11)	0.0009 (11)
O4	0.0453 (11)	0.0564 (12)	0.0540 (11)	0.0051 (9)	0.0069 (9)	−0.0139 (9)
N4	0.0321 (11)	0.0354 (11)	0.0456 (12)	−0.0013 (9)	0.0040 (9)	−0.0003 (9)
N5	0.0318 (11)	0.0397 (12)	0.0528 (13)	−0.0015 (9)	−0.0021 (10)	−0.0044 (10)
N6	0.0360 (12)	0.0512 (13)	0.0410 (12)	0.0003 (10)	−0.0020 (10)	−0.0108 (10)

### Geometric parameters (Å, º)

**Table:**

C1—C2	1.390 (4)	C24—C25	1.390 (4)
C1—C6	1.510 (4)	C24—C29	1.509 (4)
C1—N1	1.340 (3)	C24—N4	1.343 (3)
C2—C3	1.387 (4)	C25—C26	1.377 (4)
C2—H21	0.942	C25—H251	0.939
C3—C4	1.376 (4)	C26—C27	1.375 (4)
C3—H31	0.948	C26—H261	0.950
C4—C5	1.385 (4)	C27—C28	1.385 (4)
C4—H41	0.944	C27—H271	0.923
C5—C15	1.502 (4)	C28—C38	1.498 (4)
C5—N1	1.349 (3)	C28—N4	1.344 (3)
C6—O1	1.233 (3)	C29—O3	1.240 (3)
C6—N2	1.352 (3)	C29—N5	1.349 (3)
C7—C8	1.502 (4)	C30—C31	1.513 (4)
C7—N2	1.475 (3)	C30—N5	1.482 (3)
C7—H71	0.987	C30—H301	0.992
C7—H72	0.983	C30—H302	0.975
C8—H83	0.971	C31—H311	0.971
C8—H82	0.975	C31—H313	0.972
C8—H81	0.966	C31—H312	0.985
C9—C10	1.389 (4)	C32—C33	1.375 (4)
C9—C14	1.384 (4)	C32—C37	1.392 (4)
C9—N2	1.438 (3)	C32—N5	1.436 (3)
C10—C11	1.384 (4)	C33—C34	1.392 (4)
C10—H101	0.952	C33—H331	0.924
C11—C12	1.380 (5)	C34—C35	1.368 (5)
C11—H111	0.943	C34—H341	0.944
C12—C13	1.374 (4)	C35—C36	1.376 (5)
C12—H121	0.938	C35—H351	0.942
C13—C14	1.384 (4)	C36—C37	1.379 (4)
C13—H131	0.955	C36—H361	0.930
C14—H141	0.952	C37—H371	0.953
C15—O2	1.235 (3)	C38—O4	1.229 (3)
C15—N3	1.357 (3)	C38—N6	1.364 (3)
C16—C17	1.502 (4)	C39—C40	1.507 (4)
C16—N3	1.480 (3)	C39—N6	1.481 (3)
C16—H161	0.983	C39—H391	0.970
C16—H162	0.978	C39—H392	0.975
C17—H173	0.981	C40—H403	0.992
C17—H172	0.970	C40—H402	0.985
C17—H171	0.972	C40—H401	0.982
C18—C19	1.379 (4)	C41—C42	1.387 (4)
C18—C23	1.373 (4)	C41—C46	1.383 (4)
C18—N3	1.444 (3)	C41—N6	1.428 (3)
C19—C20	1.388 (4)	C42—C43	1.383 (4)
C19—H191	0.949	C42—H421	0.936
C20—C21	1.376 (5)	C43—C44	1.376 (5)
C20—H201	0.939	C43—H431	0.950
C21—C22	1.372 (5)	C44—C45	1.388 (5)
C21—H211	0.935	C44—H441	0.947
C22—C23	1.377 (4)	C45—C46	1.384 (4)
C22—H221	0.949	C45—H451	0.944
C23—H231	0.937	C46—H461	0.938
			
C2—C1—C6	117.6 (2)	C25—C24—C29	119.5 (2)
C2—C1—N1	123.1 (2)	C25—C24—N4	123.2 (2)
C6—C1—N1	119.1 (2)	C29—C24—N4	117.1 (2)
C1—C2—C3	119.0 (3)	C24—C25—C26	118.5 (3)
C1—C2—H21	120.2	C24—C25—H251	120.9
C3—C2—H21	120.8	C26—C25—H251	120.6
C2—C3—C4	118.3 (2)	C25—C26—C27	119.3 (3)
C2—C3—H31	122.4	C25—C26—H261	120.3
C4—C3—H31	119.3	C27—C26—H261	120.5
C3—C4—C5	119.4 (2)	C26—C27—C28	118.8 (3)
C3—C4—H41	120.5	C26—C27—H271	120.0
C5—C4—H41	120.1	C28—C27—H271	121.2
C4—C5—C15	122.5 (2)	C27—C28—C38	122.1 (2)
C4—C5—N1	123.0 (2)	C27—C28—N4	123.1 (2)
C15—C5—N1	114.1 (2)	C38—C28—N4	114.5 (2)
C1—C6—O1	117.9 (2)	C24—C29—O3	118.3 (2)
C1—C6—N2	119.4 (2)	C24—C29—N5	118.8 (2)
O1—C6—N2	122.7 (2)	O3—C29—N5	122.8 (2)
C8—C7—N2	112.2 (2)	C31—C30—N5	111.6 (2)
C8—C7—H71	107.9	C31—C30—H301	108.9
N2—C7—H71	107.6	N5—C30—H301	109.0
C8—C7—H72	110.0	C31—C30—H302	110.6
N2—C7—H72	107.5	N5—C30—H302	109.1
H71—C7—H72	111.6	H301—C30—H302	107.6
C7—C8—H83	110.9	C30—C31—H311	109.5
C7—C8—H82	108.6	C30—C31—H313	109.2
H83—C8—H82	108.5	H311—C31—H313	109.0
C7—C8—H81	110.5	C30—C31—H312	110.0
H83—C8—H81	109.9	H311—C31—H312	109.8
H82—C8—H81	108.4	H313—C31—H312	109.2
C10—C9—C14	120.0 (3)	C33—C32—C37	119.8 (3)
C10—C9—N2	119.4 (2)	C33—C32—N5	120.7 (2)
C14—C9—N2	120.6 (2)	C37—C32—N5	119.5 (2)
C9—C10—C11	119.5 (3)	C32—C33—C34	120.3 (3)
C9—C10—H101	120.5	C32—C33—H331	119.2
C11—C10—H101	120.0	C34—C33—H331	120.4
C10—C11—C12	120.6 (3)	C33—C34—C35	119.5 (3)
C10—C11—H111	120.4	C33—C34—H341	119.0
C12—C11—H111	119.0	C35—C34—H341	121.5
C11—C12—C13	119.5 (3)	C34—C35—C36	120.5 (3)
C11—C12—H121	120.9	C34—C35—H351	119.6
C13—C12—H121	119.6	C36—C35—H351	119.9
C12—C13—C14	120.8 (3)	C35—C36—C37	120.4 (3)
C12—C13—H131	120.8	C35—C36—H361	120.0
C14—C13—H131	118.4	C37—C36—H361	119.7
C13—C14—C9	119.5 (3)	C32—C37—C36	119.4 (3)
C13—C14—H141	121.2	C32—C37—H371	120.3
C9—C14—H141	119.3	C36—C37—H371	120.2
C5—C15—O2	119.2 (2)	C28—C38—O4	119.6 (2)
C5—C15—N3	119.6 (2)	C28—C38—N6	118.8 (2)
O2—C15—N3	121.1 (2)	O4—C38—N6	121.5 (2)
C17—C16—N3	111.8 (2)	C40—C39—N6	112.8 (2)
C17—C16—H161	109.0	C40—C39—H391	111.7
N3—C16—H161	109.7	N6—C39—H391	108.6
C17—C16—H162	108.6	C40—C39—H392	110.7
N3—C16—H162	108.4	N6—C39—H392	107.7
H161—C16—H162	109.2	H391—C39—H392	105.0
C16—C17—H173	110.4	C39—C40—H403	109.2
C16—C17—H172	110.8	C39—C40—H402	109.3
H173—C17—H172	108.5	H403—C40—H402	109.6
C16—C17—H171	109.1	C39—C40—H401	109.3
H173—C17—H171	108.8	H403—C40—H401	110.3
H172—C17—H171	109.2	H402—C40—H401	109.2
C19—C18—C23	120.4 (3)	C42—C41—C46	119.9 (3)
C19—C18—N3	120.9 (2)	C42—C41—N6	119.7 (2)
C23—C18—N3	118.6 (2)	C46—C41—N6	120.3 (2)
C18—C19—C20	118.8 (3)	C41—C42—C43	120.6 (3)
C18—C19—H191	120.5	C41—C42—H421	119.2
C20—C19—H191	120.7	C43—C42—H421	120.3
C19—C20—C21	120.9 (3)	C42—C43—C44	119.7 (3)
C19—C20—H201	119.2	C42—C43—H431	121.1
C21—C20—H201	119.8	C44—C43—H431	119.2
C20—C21—C22	119.3 (3)	C43—C44—C45	119.9 (3)
C20—C21—H211	120.4	C43—C44—H441	119.9
C22—C21—H211	120.4	C45—C44—H441	120.3
C21—C22—C23	120.5 (3)	C44—C45—C46	120.6 (3)
C21—C22—H221	119.6	C44—C45—H451	119.8
C23—C22—H221	119.9	C46—C45—H451	119.6
C22—C23—C18	120.0 (3)	C45—C46—C41	119.4 (3)
C22—C23—H231	120.1	C45—C46—H461	120.5
C18—C23—H231	119.9	C41—C46—H461	120.2
C16—N3—C18	117.2 (2)	C28—N4—C24	117.1 (2)
C16—N3—C15	118.9 (2)	C30—N5—C32	117.3 (2)
C18—N3—C15	123.4 (2)	C30—N5—C29	118.7 (2)
C5—N1—C1	117.1 (2)	C32—N5—C29	123.1 (2)
C7—N2—C9	118.0 (2)	C39—N6—C41	117.7 (2)
C7—N2—C6	118.2 (2)	C39—N6—C38	118.1 (2)
C9—N2—C6	123.5 (2)	C41—N6—C38	124.1 (2)
			
C2—C1—N1—C5	−2.0 (4)	C25—C24—N4—C28	2.0 (4)
C6—C1—N1—C5	−176.5 (2)	C29—C24—N4—C28	176.6 (2)
N1—C1—C2—C3	2.5 (4)	N4—C24—C25—C26	−1.8 (4)
C6—C1—C2—C3	177.1 (2)	C29—C24—C25—C26	−176.3 (2)
N1—C1—C6—O1	134.1 (3)	N4—C24—C29—O3	−132.6 (3)
N1—C1—C6—N2	−44.9 (3)	N4—C24—C29—N5	43.5 (3)
C2—C1—C6—O1	−40.8 (4)	C25—C24—C29—O3	42.3 (4)
C2—C1—C6—N2	140.3 (3)	C25—C24—C29—N5	−141.7 (3)
C1—C2—C3—C4	−0.5 (4)	C24—C25—C26—C27	0.4 (4)
C2—C3—C4—C5	−1.8 (4)	C25—C26—C27—C28	0.7 (4)
C3—C4—C5—N1	2.4 (4)	C26—C27—C28—N4	−0.5 (4)
C3—C4—C5—C15	−169.7 (2)	C26—C27—C28—C38	173.4 (2)
C4—C5—N1—C1	−0.5 (4)	C27—C28—N4—C24	−0.8 (4)
C15—C5—N1—C1	172.2 (2)	C38—C28—N4—C24	−175.2 (2)
N1—C5—C15—O2	−46.8 (3)	N4—C28—C38—O4	51.2 (3)
N1—C5—C15—N3	136.0 (2)	N4—C28—C38—N6	−131.1 (2)
C4—C5—C15—O2	125.9 (3)	C27—C28—C38—O4	−123.2 (3)
C4—C5—C15—N3	−51.2 (3)	C27—C28—C38—N6	54.5 (3)
O1—C6—N2—C7	−4.1 (4)	O3—C29—N5—C30	3.4 (4)
O1—C6—N2—C9	169.7 (2)	O3—C29—N5—C32	−165.3 (3)
C1—C6—N2—C7	174.8 (2)	C24—C29—N5—C30	−172.4 (2)
C1—C6—N2—C9	−11.4 (4)	C24—C29—N5—C32	18.9 (4)
C8—C7—N2—C6	96.7 (3)	C31—C30—N5—C29	−90.4 (3)
C8—C7—N2—C9	−77.4 (3)	C31—C30—N5—C32	78.9 (3)
C10—C9—N2—C6	130.0 (3)	C33—C32—N5—C29	53.8 (4)
C10—C9—N2—C7	−56.2 (3)	C33—C32—N5—C30	−115.1 (3)
C14—C9—N2—C6	−51.7 (4)	C37—C32—N5—C29	−128.1 (3)
C14—C9—N2—C7	122.1 (3)	C37—C32—N5—C30	63.1 (3)
N2—C9—C10—C11	−178.6 (2)	N5—C32—C33—C34	−179.0 (2)
C14—C9—C10—C11	3.1 (4)	C37—C32—C33—C34	2.8 (4)
N2—C9—C14—C13	178.3 (2)	N5—C32—C37—C36	178.4 (2)
C10—C9—C14—C13	−3.4 (4)	C33—C32—C37—C36	−3.4 (4)
C9—C10—C11—C12	−0.5 (4)	C32—C33—C34—C35	−0.3 (4)
C10—C11—C12—C13	−1.6 (4)	C33—C34—C35—C36	−1.7 (4)
C11—C12—C13—C14	1.3 (4)	C34—C35—C36—C37	1.0 (5)
C12—C13—C14—C9	1.2 (4)	C35—C36—C37—C32	1.6 (4)
O2—C15—N3—C16	0.6 (4)	O4—C38—N6—C39	4.1 (4)
O2—C15—N3—C18	172.3 (2)	O4—C38—N6—C41	−171.5 (2)
C5—C15—N3—C16	177.8 (2)	C28—C38—N6—C39	−173.7 (2)
C5—C15—N3—C18	−10.6 (4)	C28—C38—N6—C41	10.7 (4)
C17—C16—N3—C15	86.6 (3)	C40—C39—N6—C38	79.6 (3)
C17—C16—N3—C18	−85.7 (3)	C40—C39—N6—C41	−104.5 (3)
C19—C18—N3—C15	−60.3 (3)	C42—C41—N6—C38	−130.0 (3)
C19—C18—N3—C16	111.5 (3)	C42—C41—N6—C39	54.3 (3)
C23—C18—N3—C15	122.4 (3)	C46—C41—N6—C38	54.1 (3)
C23—C18—N3—C16	−65.8 (3)	C46—C41—N6—C39	−121.5 (3)
N3—C18—C19—C20	−178.3 (3)	N6—C41—C42—C43	−176.7 (3)
C23—C18—C19—C20	−1.0 (4)	C46—C41—C42—C43	−0.8 (4)
N3—C18—C23—C22	177.7 (3)	N6—C41—C46—C45	177.9 (3)
C19—C18—C23—C22	0.4 (4)	C42—C41—C46—C45	2.1 (4)
C18—C19—C20—C21	1.2 (5)	C41—C42—C43—C44	−0.6 (5)
C19—C20—C21—C22	−0.7 (5)	C42—C43—C44—C45	0.7 (5)
C20—C21—C22—C23	0.0 (5)	C43—C44—C45—C46	0.6 (5)
C21—C22—C23—C18	0.2 (5)	C44—C45—C46—C41	−2.0 (5)
N1—C1—C2—H21	−178	N4—C24—C25—H251	178
C6—C1—C2—H21	−4	C29—C24—C25—H251	3
C1—C2—C3—H31	179	C24—C25—C26—H261	−179
H21—C2—C3—C4	−180	H251—C25—C26—C27	−179
H21—C2—C3—H31	0	H251—C25—C26—H261	1
C2—C3—C4—H41	−179	C25—C26—C27—H271	−180
H31—C3—C4—C5	179	H261—C26—C27—C28	−179
H31—C3—C4—H41	1	H261—C26—C27—H271	0
H41—C4—C5—N1	180	H271—C27—C28—N4	180
H41—C4—C5—C15	8	H271—C27—C28—C38	−6
H71—C7—N2—C6	−145	H301—C30—N5—C29	149
H71—C7—N2—C9	41	H301—C30—N5—C32	−41
H72—C7—N2—C6	−24	H302—C30—N5—C29	32
H72—C7—N2—C9	161	H302—C30—N5—C32	−159
N2—C7—C8—H81	−62	N5—C30—C31—H311	179
N2—C7—C8—H82	57	N5—C30—C31—H312	58
N2—C7—C8—H83	176	N5—C30—C31—H313	−62
H71—C7—C8—H81	180	H301—C30—C31—H311	−61
H71—C7—C8—H82	−62	H301—C30—C31—H312	178
H71—C7—C8—H83	58	H301—C30—C31—H313	59
H72—C7—C8—H81	58	H302—C30—C31—H311	57
H72—C7—C8—H82	177	H302—C30—C31—H312	−63
H72—C7—C8—H83	−64	H302—C30—C31—H313	177
N2—C9—C10—H101	0	N5—C32—C33—H331	0
C14—C9—C10—H101	−179	C37—C32—C33—H331	−178
N2—C9—C14—H141	−1	N5—C32—C37—H371	−1
C10—C9—C14—H141	177	C33—C32—C37—H371	178
C9—C10—C11—H111	178	C32—C33—C34—H341	−179
H101—C10—C11—C12	−179	H331—C33—C34—C35	−179
H101—C10—C11—H111	−1	H331—C33—C34—H341	2
C10—C11—C12—H121	180	C33—C34—C35—H351	−180
H111—C11—C12—C13	−180	H341—C34—C35—C36	178
H111—C11—C12—H121	2	H341—C34—C35—H351	−1
C11—C12—C13—H131	−178	C34—C35—C36—H361	−178
H121—C12—C13—C14	180	H351—C35—C36—C37	179
H121—C12—C13—H131	1	H351—C35—C36—H361	0
C12—C13—C14—H141	−179	C35—C36—C37—H371	−179
H131—C13—C14—C9	−179	H361—C36—C37—C32	−179
H131—C13—C14—H141	0	H361—C36—C37—H371	0
H161—C16—N3—C15	−35	H391—C39—N6—C38	−45
H161—C16—N3—C18	153	H391—C39—N6—C41	131
H162—C16—N3—C15	−154	H392—C39—N6—C38	−158
H162—C16—N3—C18	34	H392—C39—N6—C41	18
N3—C16—C17—H171	55	N6—C39—C40—H401	52
N3—C16—C17—H172	−65	N6—C39—C40—H402	−67
N3—C16—C17—H173	174	N6—C39—C40—H403	173
H161—C16—C17—H171	176	H391—C39—C40—H401	175
H161—C16—C17—H172	56	H391—C39—C40—H402	55
H161—C16—C17—H173	−64	H391—C39—C40—H403	−65
H162—C16—C17—H171	−65	H392—C39—C40—H401	−69
H162—C16—C17—H172	175	H392—C39—C40—H402	172
H162—C16—C17—H173	55	H392—C39—C40—H403	52
N3—C18—C19—H191	2	N6—C41—C42—H421	4
C23—C18—C19—H191	179	C46—C41—C42—H421	180
N3—C18—C23—H231	−1	N6—C41—C46—H461	−2
C19—C18—C23—H231	−179	C42—C41—C46—H461	−178
C18—C19—C20—H201	−178	C41—C42—C43—H431	177
H191—C19—C20—C21	−179	H421—C42—C43—C44	179
H191—C19—C20—H201	2	H421—C42—C43—H431	−4
C19—C20—C21—H211	179	C42—C43—C44—H441	−179
H201—C20—C21—C22	178	H431—C43—C44—C45	−177
H201—C20—C21—H211	−2	H431—C43—C44—H441	3
C20—C21—C22—H221	179	C43—C44—C45—H451	−179
H211—C21—C22—C23	−180	H441—C44—C45—C46	−180
H211—C21—C22—H221	−1	H441—C44—C45—H451	0
C21—C22—C23—H231	179	C44—C45—C46—H461	178
H221—C22—C23—C18	−179	H451—C45—C46—C41	178
H221—C22—C23—H231	0	H451—C45—C46—H461	−2

### Hydrogen-bond geometry (Å, º)

**Table:**

*D*—H···*A*	*D*—H	H···*A*	*D*···*A*	*D*—H···*A*
C34—H341···O1^i^	0.94	2.42	3.319 (4)	159
C42—H421···O2^ii^	0.94	2.43	3.271 (4)	150
C7—H71···O2^ii^	0.99	2.40	3.208 (4)	138
C43—H431···O3^iii^	0.95	2.66	3.443 (4)	140
C30—H301···O4^iv^	0.99	2.36	3.237 (4)	147
C23—H231···O4^v^	0.94	2.60	3.125 (3)	116

Symmetry codes: (i) −*x*+1, −*y*+1, −*z*+1; (ii) −*x*+1, −*y*+1, −*z*+2; (iii) *x*+1, *y*, *z*; (iv) −*x*, −*y*+2, −*z*+1; (v) −*x*, −*y*+1, −*z*+2.
